# Digital photography in skin cancer screening by mobile units in remote areas of Brazil

**DOI:** 10.1186/s12895-014-0019-1

**Published:** 2014-12-24

**Authors:** Carlos Eduardo Goulart Silveira, Thiago Buosi Silva, José Humberto Guerreiro Tavares Fregnani, René Aloisio da Costa Vieira, Raphael Luiz Haikel, Kari Syrjänen, André Lopes Carvalho, Edmundo Carvalho Mauad

**Affiliations:** Department of Cancer Prevention, Barretos Cancer Hospital, Rua Antenor Duarte Villella, 1331 - Bairro Dr. Paulo Prata, 14784-400 Barretos, SP Brazil; Department of Surgery, Barretos Cancer Hospital, Barretos, SP Brazil; Teaching and Research Institute, Barretos Cancer Hospital, Barretos, SP Brazil; Department of Oncology & Radiotherapy, Turku University Hospital, Turku, Finland

**Keywords:** Teledermatology, Skin cancer, Diagnosis, Public health, Accuracy, Verification bias

## Abstract

**Background:**

Non-melanoma skin cancer (NMSC) is one of the most common neoplasms in the world. Despite the low mortality rates, NMSC can still cause severe sequelae when diagnosed at advanced stages. Malignant melanoma, the third most common type of skin cancer, has more aggressive behavior and a worse prognosis. Teledermatology provides a new tool for monitoring skin cancer, especially in countries with a large area and unequal population distribution.

This study sought to evaluate the performance of digital photography in skin cancer diagnosis in remote areas of Brazil.

**Methods:**

A physician in a Mobile Prevention Unit (MPU) took four hundred sixteen digital images of suspicious lesions between April 2010 and July 2011. All of the photographs were electronically sent to two oncologists at Barretos Cancer Hospital who blindly evaluated the images and provided a diagnosis (benign or malignant). The absolute agreement rates between the diagnoses made by direct visual inspection (by the MPU physician) and through the use of digital imaging (by the two oncologists) were calculated. The oncologists’ accuracy in predicting skin cancer using digital imaging was assessed by means of overall accuracy (correct classification rate), sensitivity, specificity and predictive value (positive and negative). A skin biopsy was considered the gold standard.

**Results:**

Oncologist #1 classified 59 lesions as benign with the digital images, while oncologist #2 classified 27 lesions as benign using the same images. The absolute agreement rates with direct visual inspection were 85.8% for oncologist #1 (95% CI: 77.1-95.2) and 93.5% for oncologist #2 (95% CI: 84.5-100.0). The overall accuracy of the two oncologists did not differ significantly.

**Conclusions:**

Given the high sensitivity and PPV, Teledermatology seems to be a suitable tool for skin cancer screening by MPU in remote areas of Brazil.

## Background

Skin cancer is the most common malignancy in many parts of the world, including Brazil [1,2]. More than 2 million new non-melanoma skin cancer (NMSC) cases are diagnosed in the United States each year [3], and approximately 76,250 new cases of malignant melanoma were expected to be diagnosed in 2012 [4]. However, the true incidence of NMSC remains unknown because these lesions are not commonly reported to cancer registries. It has been estimated that 25% of all new cancer cases diagnosed in Brazil in 2012 will be skin cancers, with approximately 134,170 new cases of NMSC and 6,230 malignant melanomas expected to be identified. These numbers represent an approximately 16% increase in new NMSC cases and a 4% increase in melanomas compared with 4 years ago [1].

Despite a low mortality rate, NMSC can still cause severe sequelae when diagnosed at advanced stages [5] because these lesions occur predominantly on sun-exposed areas such as the face, which can become disfigured. Moreover, significant morbidity costs may occur [6]. Malignant melanoma, the third most common type of skin cancer, has more aggressive behavior and a worse prognosis, causing a significant decrease in life expectancy and lost productivity [6].

Currently, the best method for the early detection of skin cancer is to identify changes in skin lesions, including the appearance of new growths [4]. However, this strategy is not easily implemented in developing countries due to a shortage of trained professionals and their availability in remote areas. For example, in the State of São Paulo, which has the largest cancer registry in Brazil, 9% of basal cell cancer (BCC) cases, 21% of squamous cell cancer (SCC) cases and 49% of malignant melanomas are diagnosed at stages II, III or IV [7].

Teledermatology provides a new tool for monitoring skin cancer in countries such as Brazil, which has a large area and an unequal population distribution. Teledermatology essentially involves sending digital images to specialized cancer centers for evaluation by trained experts [8-10] and provides a platform for professional training programs and physicians to discuss complex cases without the necessity of transporting patients, which may lead to substantial savings in time and cost [11]. This approach also effectively reduces the waiting times for surgical treatment [12] and facilitates the spread of dermatological knowledge into poor regions of the world [13].

Because of the high incidence of skin cancer in Brazil, the difficulty accessing doctors in poor areas and the lack of studies on teledermatology in developing countries, we decided to utilize the existing telecommunication technology and evaluate the accuracy of digital imaging in diagnosing skin cancer in remote areas of Brazil.

## Methods

### Patients

The skin cancer screening was performed by a Mobile Prevention Unit (MPU) of Barretos Cancer Hospital (BCH). The MPU regularly visits the remote areas of Brazil, including the states of Mato Grosso, Mato Grosso do Sul and Rondônia, screening the local people for prostate, cervical and skin cancers. The MPU trailer is fully equipped to perform clinical procedures and ambulatory surgeries. The MPU team consists of a physician, a nurse, three nurse technicians and a driver. This team is able to perform 40 clinical dermatology examinations or procedures per day, including cryotherapy and surgery. All of the patients examined at the MPU were previously screened by a nurse from the local municipality who was trained at BCH. A more detailed description of the MPU concept has been published elsewhere [14].

The present study included individuals with skin lesions that were determined to be suspicious after a direct visual inspection by a physician between April 2010 and July 2011. These patients were evaluated in the MPU, and their lesions were photographed (digital imaging). All of the lesions suspected to be malignant by the MPU physician were biopsied and/or excised after photography and submitted to the Department of Pathology at BCH for histological evaluation. The Institutional Review Board (IRB) at BCH previously approved the research protocol (No. 377/2010). All of the subjects signed an informed consent agreement.

### Methods

The lesions were photographed by the MPU physician using a Sony Cybershot DSC-5780 digital camera with 8.1-megapixel resolution. One digital image of each lesion was taken at a distance of 60 cm to evaluate the lesion topography, with an additional image taken at a shorter distance (using the macro mode) to evaluate the lesion details.

Pertinent information such as age, skin complexion, location of the lesion, stage and pathology results were collected and used for the TNM Classification of Malignant Tumors (AJCC) system, 7th edition [15]. All of the diagnoses were classified according to the International Classification of Diseases (ICD-10). We used the Skin Type (or complexion) Classification System proposed by Fitzpatrick, which utilizes the Skin Type 1–6 scale where 1 denotes pale white skin, 2 denotes white skin, 3 denotes light brown skin, 4 denotes moderate brown skin, 5 denotes dark brown skin and 6 denotes pigmented dark brown to black skin [16].

All of the digital images were coded, stored and submitted at random to two oncologists at BCH. These two experts were blinded to the MPU physician’s diagnosis and pathology reports, and classified the images using the following options: 1) a malignant lesion, oncological treatment is indicated; 2) a benign lesion, no treatment required; 3) unknown; or 4) a low-quality image. Both the oncologists and the MPU physician have more than 10-years of experience in skin cancer screening.

### Statistical analysis

The diagnoses made by all three physicians were characterized by descriptive statistics using SPSS for Windows software (v. 17.0, SPSS Inc., Chicago, IL, USA). The absolute agreement rates between the diagnoses obtained from direct visual inspection (by the MPU physician) and through the use of digital images (by the two oncologists) were calculated. The oncologists’ performance in predicting skin cancer using the digital images was evaluated on the basis of overall accuracy (correct classification rate), sensitivity, specificity, positive predictive value (PPV) and negative predictive value (NPV). The result of a skin biopsy was considered the gold standard. Confidence intervals (95% CI) were calculated whenever appropriate.

## Results

A total of 2,592 patients underwent dermatological examination at the MPU throughout the duration of the study. Of these, 460 (17.7%) had a suspicious lesion that was classified as possibly malignant by the MPU physician. These lesions were imaged, biopsied/removed and submitted for histopathological examination at BCH. Of the 460 patients, 21 (4.6%) were excluded from the study because of poor quality photos, leaving 439 patients with pathological results. Of these 439 lesions, 23 (5.2%) were excluded because of incomplete data preventing the identification of the patient, while 364 (87.5%) were confirmed to be malignant by the biopsy. The majority of the lesions were BCCs (78.5%), and most were located in the head and neck area (75%). A large majority of the lesions (93%) were classified as stages 0 and I (Table 1). These patients had a mean age of 63.5 years (range: 19 to 93 years) and were from 5 states of Brazil (MT, MS, RO, GO and MG). The patients were predominantly (81%) light-skinned, i.e., skin type 1 or 2 (Table 1).

**Table 1:** **Diagnosis and location of the biopsied or removed lesions and skin complexion of the patients**

**Variable**	**Category**	**N (%)**
Pathology*	Basal cell carcinoma	286 (78.5)
	Squamous cell carcinoma	59 (16.2)
	Melanoma	5 (1.4)
	Other**	14 (3.8)
Site	Head and Neck	273 (75.0)
	Trunk	28 (7.6)
	Upper limbs	61 (16.7)
	Lower limbs	2 (0.5)
Clinical stage***	0	11 (3.5)
	I	280 (89.5)
	II	21 (6.7)
	III	1 (0.3)
	IV	0 (0)
Skin type scale	1-2	295 (81)
	3-4	69 (19)
	5-6	0 (0)

*52 lesions were confirmed to be benign by the biopsy; **Bowen’s disease, malignant trichoepithelioma and metatypical carcinoma; ***51 patients were not staged because they did not go to BCH for diagnostic follow-up.

Altogether, 416 digital images were electronically sent the two oncologists, who completely blinded by any clinical description or attached information. The oncologists classified the tumors in the images as either malignant or benign. Oncologist #1 classified 59 lesions as benign using the digital images, while oncologist #2 classified 27 lesions as benign using the same images., The absolute agreement rates with the direct visual inspection were 85.8% for oncologist #1 (95% CI: 77.1-95.2) and 93.5% for oncologist #2 (95% CI: 84.5-100.0) (Table 2). These rates were not statistically different.

**Table 2:** **Number and percentage of lesions, as determined by the raters and the skin biopsy**

**Physician**	**Physician’s diagnosis**	**Skin biopsy**		**Total**
		**Malignant**	**Benign**	
		**N (%)**	**N (%)**	
**MPU physician**	Malignant	364 (87.5)	52 (12.5)	416
	Benign	-	-	-
**Oncologist #1**	Malignant	325 (91.0)	32 (9.0)	367
	Benign	39 (66.1)	20 (33.9)	59
**Oncologist #2**	Malignant	350 (90.0)	39 (10.0)	389
	Benign	14 (51.9)	13 (48.1)	27

MPU = mobile prevention unit.

Table 3 summarizes the oncologists’ performance in predicting skin cancer using the digital images. The overall accuracy, specificity and predictive values (negative and positive) did not differ significantly between the two oncologists; however, oncologist #2 had a slightly, but significant, higher sensitivity.

**Table 3:** **Oncologists’ performance indicators in predicting skin malignancy using digital imaging**

	**Oncologist #1**	**Oncologist #2**
	**% (95% CI)**	**% (95% CI)**
**Sensitivity**	89.3 (85.7-92.3)	96.2 (93.6-97.9)
**Specificity**	38.5 (25.3-53.0)	25.0 (14.0-39.0)
**Positive predictive value**	91.0 (87.6-93.8)	90.0 (86.6-92.8)
**Negative predictive value**	33.9 (22.1-47.4)	48.2 (26.7-68.1)
**Overall accuracy**	85.3 (76.7-94.7)	87.3 (78.5-96.7)

## Discussion

The most remote areas of Brazil are located North of the Tropic of Capricorn and near the Equator—areas where solar radiation is very intense [17]. Since the 1980s, these regions have received large numbers of migrants from the Southern and Midwestern regions of Brazil, where people are predominantly of European descent. Thus, the majority of these inhabitants have light skin. Not surprisingly, this migration has increased the incidence of skin cancer in this area [1]. Despite this increase, the Brazilian government is reluctant to invest in permanent programs for skin cancer prevention because of the existing controversy over whether or not screening increases the survival of melanoma patients [18].

Developing countries exhibit an unequal distribution of doctors throughout their territories. In Brazil, the concentration of physicians is higher in metropolitan areas, and there is a huge gap in the availability of these professionals in remote areas [19]. Brazil averages one doctor for every 551 inhabitants [20], with this number varying from one doctor for every 232 inhabitants in the city of São Paulo to one doctor for every 10,000 inhabitants in some remote areas of the Amazon States and Rondônia [20,21]. This situation is further exacerbated when medical experts, such as dermatologists, are sought. In this case, there may be one dermatologist for every 90,000 inhabitants in certain regions of Brazil [21,22].

Given this reality, one alternative is teledermatology, which is being promoted worldwide as an effective tool for diagnosing skin cancer in remote areas [13]. This method reduces the waiting times for these patients and results in good customer satisfaction scores [23,24], satisfactory clinical results [25] and good cost-effectiveness [26]. Despite the fact that teledermatology has been recognized as an important skin cancer screening tool in even large populations [27], few studies have investigated the use of teledermatology for diagnosing skin cancer [28,29]. This technique could be particularly useful in developing countries [13], where skin cancer predominantly presents in the clinic at advanced stages. To our knowledge, this is the first study to use mobile units for teledermatology.

The present study was a store-and-forward teledermatology study used for skin cancer screening. The study was performed using an MPU in which a physician examined a large number of patients who were referred because of a suspected skin cancer lesion. These patients were subjected to a biopsy and/or lesion removal for histopathological confirmation. Thus, the present study represents a typical screening setting, where a positive test (classification as suspicious by digital image) was verified by the gold standard (skin biopsy). This study showed a high rate of agreement between the MPU physician’s diagnosis by visual inspection and the diagnosis given by the two oncologists using the digital images as a diagnostic tool. This result is quite impressive given the fact that oncologists #1 and #2 performed their analysis in a tertiary cancer hospital (BCH) up to 1,200 miles away from the MPU. These concordance values are even more impressive if one also considers that some of this variability may be due to differences in data interpretation between the physicians and the different experiences of the physicians involved and not due to the technology itself [13]. It seems that the physician’s experience in skin cancer screening may be more important than the technology, a finding that is not surprising. Previous reports have found that inter-observer agreement varies significantly depending on case selection, the use of classification criteria [30] and the level of expertise of the physicians [13,28]. Thus, the high level of concordance between the oncologists in this study may also be related to the fact that they have more than 10 years of experience in skin cancer screening in Brazil.

When the diagnoses of the physicians were translated to performance indicators of digital imaging, the crude sensitivity for oncologist #1 was 89.3% and the crude sensitivity for oncologist #2 was even higher at 96.2% (Table 3). The PPV was equally high for both physicians, but the specificity and NPV were not particularly impressive. It must be emphasized that these calculations are based on incomplete evaluations [31], as biopsy verification was only performed for test-positive cases, i.e., the digital images classified as malignant. This situation occurred because it would be unethical to perform a biopsy on normal skin tissue. For any type of screening, the optimal test is the one with the highest PPV [31]. In this respect, the teledermatology setting tested here seems to be a highly suitable screening tool.

From the clinical point of view, the type of lesions missed by this screening approach is important; for example, missing a malignant melanoma is more serious than missing a BCC or SCC, which exhibit a substantially more protracted clinical course. According to our histological records, there were 5 histologically-confirmed malignant melanomas in the present series. Of those malignancies, observer 1 diagnosed 1/5 correctly, while observer 2 correctly diagnosed 3/5 cases. This result would justify using dermatoscopy to screen all pigmented lesions. However, this practice is not feasible in large countries with limited resources in rural areas, such as Brazil. A dermatoscope is an expensive piece of equipment, and the proper use of this technique necessitates the practical training of the physicians, precluding the use of this instrument in population-based screening for skin cancer.

Digital image diagnosis approach seems to be a viable option for teledermatology settings that utilize MPUs. One limitation of this study is the fact that we were not able to capture images from a camera attached to a dermatoscope, which is known to further increase diagnostic precision [32-34]. We only used a simple digital camera, which is easier for any health professional to handle than a more complex device such as a dermatoscope. Another limitation was the lack of clinical histories for the examined patients (work in agriculture, family history of skin cancer, etc.) and a description of the characteristics of the lesion (raised or flat, lesion size, time of development, etc.) to assist the observing physicians in making the final diagnoses.

Despite the limitations of this study, the high number of evaluated lesions and the high concordance between the observers clearly indicate that teledermatology could be used as an effective tool to screen for malignant skin lesions, especially in the remote areas of developing countries.

## Conclusion

This study showed high agreement between direct visual inspection and digital imaging in identifying suspicious skin lesions. Moreover, digital imaging played a previously unrecognized role in predicting skin cancer. Hence, this study suggests that it is feasible to use digital imaging as a tool to screen for skin cancer in a population-based setting and that this approach would be particularly useful in remote areas.
